# LINC00511 promotes gastric cancer progression by regulating SOX4 and epigenetically repressing PTEN to activate PI3K/AKT pathway

**DOI:** 10.1111/jcmm.16656

**Published:** 2021-08-24

**Authors:** Qianwei Wang, Xiang Mao, Fen Luo, Jun Wang

**Affiliations:** ^1^ Department of General Surgery Huashan Hospital Affiliated to Fudan University Shanghai China

**Keywords:** ceRNA, gastric cancer, LINC00511

## Abstract

Gastric cancer (GC) serves as a common malignancy. Long non‐coding RNAs (lncRNAs) have been proven to regulate many cancers, including GC. Long intergenic non‐protein‐coding RNA 511 (LINC00511) has been poorly studied in GC, but its detailed regulatory mechanism has not been identified. Here, LINC00511 was detected to be highly expressed in GC cells. Functional assays were conducted and uncovered that LINC00511 boosted cell proliferation, migration, stemness and EMT process while inhibiting the apoptosis of GC cells. From a series of mechanism experiments, it was found that at the transcriptional level, LINC00511 recruited EZH2 (enhancer of zeste 2 polycomb repressive complex 2 subunit) to the promoter of PTEN (phosphatase and tensin homolog) and facilitated methylation of PTEN promoter. LINC00511 epigenetically repressed PTEN to activate the PI3K/AKT pathway. Moreover, SRY‐box transcription factor 4 (SOX4) activated the transcription of LINC00511. At the post‐transcriptional level, LINC00511 sponged miR‐195‐5p to elevate SOX4 expression in GC cells. On the whole, the present study disclosed that SOX4‐induced LINC00511 activated SOX4 via competing endogenous RNA (ceRNA) pattern and epigenetically repressed PTEN to activate PI3K/AKT pathway by recruiting EZH2, thus facilitating GC cell proliferation, migration and stemness while inhibiting GC cell apoptosis.

## 1 INTRODUCTION

GC is one of the leading causes of cancer deaths around the world, with smoking and atrophic gastritis as the common risk factors.^1^, ^2^ Although GC can be cured at early stages, for GC patients at advanced stage after receiving systemic treatment, the 5‐year survival rate is still frustratingly beneath 5%.^3^ Therefore, to explore the molecular mechanism of GC occurrence is essential for improving its treatment.

Due to the advancing achievements in whole‐genome sequencing technology, a new group of regulatory RNAs have been discovered. LncRNAs are transcribed RNA molecules longer than 200 nucleotides lacking protein‐coding capacity.^4^ Accumulating reports have revealed that lncRNAs are crucial contributors in cellular development and biological processes. Their dysregulation has also been studied in various malignancies, including GC. LncRNA MIR4435‐2HG promotes GC phenotypes through targeting desmoplakin and activating Wnt/β‐catenin pathway.^5^ DLX6‐AS1 serves as the ceRNA to up‐regulate OCT1 via sponging miR‐204‐5p and promotes GC progression and epithelial‐mesenchymal transition (EMT).^6^ LncRNA LINC00707 promotes progression of GC through interaction with protein HuR to promote mRNA stabilization of VAV3 and F11R.^7^ Among multiple putative functions of lncRNAs, it is speculated that lncRNAs can bind to enzymes modified by chromatin and consequently exert synergistic effects in the epigenetic activation or the silencing of target genes.^8^ FEZF1‐AS1 inhibits p21 expression via binding to LSD1 protein to promote GC development.^9^ HOXD‐AS1 can recruit WDR5 to epigenetically regulate the expression of target genes and influence the biological behaviours of prostate cancer.^10^ LncRNA AGAP2‐AS1 epigenetically inhibits TFPI2 expression through serving as the scaffold of EZH2 and LSD1, thus facilitating glioblastoma development.

Long intergenic non‐protein‐coding RNA 511 (LINC00511) is a familiar gene in cancer research. Based on GEPIA database, LINC00511 is up‐regulated in multiple cancers, including GC. According to previous studies, LINC00511 can target miR‐625‐5/NFIX axis and promote the proliferation of tumours. Therefore, LINC00511 can be viewed as a target for GC treatment.^11^ LINC00511 interacts with EYA1 to promote the development of hepatocellular carcinoma via mediating miR‐195, which can be treated as a therapeutic biomarker for hepatocellular carcinoma diagnosis and treatment.^12^ Also, LINC00511 promotes the progression of non‐small‐cell lung cancer through down‐regulating LATS2 and KLF2 by binding to EZH2 and LSD1.^13^ What's more, LINC00511 can enhance the proliferative and invasive ability of gastric cancer via modulating miR‐515‐5p. Therefore, it is speculated that such lncRNA may be considered as a potential target for the development of anti‐cancer drugs.^14^ However, whether there still exists some other underlying mechanism of LINC00511 in GC remains to be unveiled.

Recently, ceRNA network has attracted more and more attention in cancer research. CeRNA mechanism refers to that lncRNAs could bind to the complementary seed region of microRNAs (miRNAs) as an endogenous RNA, thus antagonizing miRNA‐mediated suppression on target genes. Ye Y et al have found that lncRNA H19 functions as a sponge adsorbing to miR‐193b. Therefore, it can protect MAPK1 from degradation caused by miR‐193b and enhance cell aggressiveness in hepatocellular carcinoma.^15^ FTL competes with lncRNA LINC00467 by binding to miR‐133b thus regulating the chemoresistance and metastasis of colorectal cancer.^16^ MALAT1 enhances ZHX1 expression by sponging miR‐199a thus affecting the progression of glioblastoma.^17^ LINC00673 acts as a ceRNA by sponging miR‐515‐5p to elevate MARK4 expression, thus promoting proliferation of breast cancer cells.^18^

PI3K/AKT1/mTOR pathway is widely reported in malignant tumours. Wang LL has put forward that lncRNA LINC01419 inhibits the development of GC cells via activating PI3K/AKT1/mTOR pathway.^19^ IL‐17 triggers the changes in the phenotypes of glioma cells through activating PI3K/AKT1/NF‐κB‐p65 pathway.^20^ What's more, YY1 can bind to the promoter region of TPPP, thus leading to phenotype changes of pancreatic cancer via inducing p38/MAPK and PI3K/AKT pathways.^21^ SOX9 serves as the transcription suppressor for miR‐203a via binding to miR‐203a promoter and then blocks miR‐203a‐mediated inhibition on PI3K/AKT/mTOR pathway in oesophageal squamous cell carcinoma.^22^ However, whether LINC00511 could activate PI3K/AKT pathway in GC cells has not been studied.

Therefore, we aimed at exploring another molecular mechanism of LINC00511 in the tumour progression of GC cells.

## 2 MATERIALS AND METHODS

### 2.1 Cell lines and culture

Human GC cell lines (AGS, HGC‐27, ACP01, SNU‐1) and human normal oesophageal epithelial cell line (Het‐1A) were procured from Cell Bank of the Chinese Academy of Sciences (Shanghai, China). RPMI 1640 medium covering 1% Pen/Strep mixture and 10% FBS was applied for cell culture under 37°C and 5% CO_2_, as requested by supplier (Invitrogen, Carlsbad, CA, USA).

### 2.2 Quantitative real‐time polymerase chain reaction (qRT‐PCR)

Total RNA extracted from cultured cells using TRIzol reagent (Invitrogen) was then reversely transcribed into cDNA using Reverse Transcription Kit (Qiagen, Hilden, Germany). Quantitation was performed using SYBR Green Master Mix (TaKaRa Bio, Otsu, Japan), with GAPDH or U6 as the internal control. Relative expression was acquired based on 2^−ΔΔCt^ method.

### 2.3 Plasmid transfection

To silence gene expression, the short hairpin RNAs (shRNAs), including sh‐LINC00511, sh‐EZH2, sh‐PTEN and sh‐SOX4, were commercially acquired from GenePharma Company (Shanghai, China). To overexpress SOX4, the full‐length cDNA sequence of SOX4 was amplified and cloned into pcDNA3.1 vector (Invitrogen), termed as pcDNA3.1‐SOX4. What's more, miR‐195‐5p, NC mimics and NC inhibitors were also procured from GenePharma. Cells of HGC‐27 and SNU‐1 were plated in 6‐well plates for 48‐hour transfection with plasmids and relevant negative control (NC) using Lipofectamine 2000 (Invitrogen).

### 2.4 EdU staining assay

After the 48‐hour plasmid transfection, HGC‐27 and SNU‐1 cells were planted on sterile coverslips in 24‐well plates and treated with EdU staining kit (RiboBio, Guangzhou, China) as per the user guide. After fixing and permeation, cell nuclei were double‐stained with EdU and DAPI (Beyotime, Shanghai, China). At last, the EdU‐positive cells were observed through laser confocal microscopy (Olympus, Tokyo, Japan).

### 2.5 Colony formation assay

As for this assay, 800 cells were obtained after 48‐hour stable transfection, then plated in 6‐well plate and cultivated in medium for 14 days to allow colony formation. After being fixed by 4% paraformaldehyde, cells were stained by 0.1% crystal violet. Cell proliferation was estimated via the number of colonies consisting of more than 50 cells.

### 2.6 TUNEL staining assay

Cell samples of HGC‐27 and SNU‐1 on coverslips were fixed for 10 minutes and permeabilized by 0.1% Triton X‐100 for 15 minutes. Apoptosis was evaluated by In Situ Cell Apoptosis Detection Kit (Roche, Basel, Switzerland) as per manual. Cell nucleus was visualized using DAPI solution, and apoptosis index was presented as the percentage of TUNEL‐positive cells to DAPI‐positive cells. Experimental procedures were repeated independently more than three times.

### 2.7 Flow cytometry analysis

After transfected for 48 hours, GC cells were gathered to the 6‐well plates and FITC Annexin V Apoptosis Detection Kit was utilized to monitor the apoptotic cells following the specification of BD Biosciences (San Jose, CA). The cell apoptosis was monitored using FACScan flow cytometry (BD Biosciences).

### 2.8 Western blot

When the cell confluence was above 80%, the cell protein lysates were prepared and separated by 12% SDS‐PAGE, shifted to PVDF membranes and then incubated in 5% skimmed milk. After washing, the diluted specific primary antibodies and HRP‐labelled secondary antibodies were used. Immunoreactive bands were finally visualized using the ECL detection method (Bio‐Rad laboratory, Hercules, CA). All of the antibodies were procured from Abcam (Cambridge, MA).

### 2.9 Transwell assay

Cells (1 × 10^5^) of HGC‐27 and SNU‐1 in serum‐free RPMI 1640 medium were planted on the top of 24‐well Transwell chambers (Corning Incorporated, Corning, NY), and complete medium was used to supply the lower chamber. Twenty‐four hours later, cells in the upper layer were removed and then fixed in methanol solution for 15 minutes. 0.1% Crystal violet was adopted for staining for 10 minutes, and the migrated cells were observed and counted under a microscope (10 × 10).

### 2.10 Immunofluorescence (IF)

HGC‐27 and SNU‐1 cells were plated on the culture slides. Forty‐eight hours after transfection, cells were fixed by 4% PFA solution at room temperature for half an hour and cells were washed using washing buffer. After blocking in 5% BSA, cells were incubated with the primary antibodies against E‐cadherin and N‐cadherin in PBS for 2 hours. Cells were then washed in PBS for incubation with secondary antibodies, then stained in DAPI solution and examined by Olympus microscopy.

### 2.11 Sphere formation

Ten treated cells of HGC‐27 and SNU‐1 in sphere medium were plated into the each well of 96‐well ultralow attachment plates (Corning) for 7‐day cell culture purposes. Sphere cells with diameter >50 mm were counted.

### 2.12 In vivo xenograft tumour assay

Male BALB/C nude mice, aged 6‐8 weeks from Shanghai SIPPR‐BK Laboratory Animal (Shanghai, China), were kept in micro‐isolator cages. GC cells with sh‐LINC00511 or sh‐NC were suspended in serum‐free culture medium and then injected subcutaneously into mice. Tumour volume was tested every 4 days. Animal model was established for 4 weeks, and then, tumours were excised and weighed. The animal‐related study protocol was approved by the Ethics Committee of Huashan Hospital Affiliated to Fudan University.

### 2.13 Immunohistochemistry (IHC)

After fixing, the tissue samples collected from in vivo xenograft tumour assay were dehydrated and embedded in paraffin. Then, the consecutive sections of 4 μm thick were prepared for IHC analysis using specific antibodies to Ki‐67, PCNA, SOX2, E‐cadherin and N‐cadherin.

### 2.14 Subcellular fraction

Cytoplasmic & Nuclear RNA Purification Kit (Norgen, Belmont, CA) was acquired for the assay in GC cells. After centrifugation, the cells were subjected to cell disruption buffer. The contents of GAPDH, PSMA3‐AS1 and U6 were separately analysed in two cell fractions. The finally isolated RNAs were examined using qRT‐PCR. Experimental procedures were repeated independently more than three times.

### 2.15 Fluorescence in situ hybridization (FISH) assay

The RNA FISH probe designed for LINC00511 was procured from RiboBio and employed for incubation with air‐dried cells in hybridization buffer as per instruction. The fixed cell samples were rinsed in PBS and then dehydrated. After that, the air‐dried cells were hybridized with FISH probe prior to DAPI staining. Three hours after hybridization, cell nucleus was stained using DAPI solution and observed by Olympus fluorescence microscope. GAPDH and U6 were treated as the cytoplasm and the nucleus control, respectively. Experiments were repeated for three times.

### 2.16 Luciferase reporter assay

For promoter assay, the PTEN promoter or LINC00511 promoter was severally amplified and inserted into luciferase reporter pGL3 vector (Promega, Madison, WI) and then transfected with indicated plasmids. Besides, the LINC00511 fragment or SOX4 fragment covering miR‐195‐5p target sites (wild‐type and mutated) was separately sub‐cloned into pmirGLO vector (Promega) and then transfected with miR‐195‐5p‐mimics or NC mimics. After two days, all luciferase intensities were examined using Luciferase Reporter Assay System (Promega).

### 2.17 Chromatin immunoprecipitation (ChIP) assay

Using ChIP kit, ChIP assay was implemented as instructed by provider (Millipore, Billerica, MA). The crosslinked chromatin was fragmented by ultrasonic for immunoprecipitation with anti‐Histone H3, anti‐SOX4, anti‐H3K27me3 or negative control anti‐IgG antibody. The finally recovered chromatin by magnetic beads was subjected to qRT‐PCR.

### 2.18 Pull‐down assays

For RNA pull‐down, Pierce Magnetic RNA‐Protein Pull‐Down Kit was used as per guidebook (Thermo Fisher Scientific, Waltham, MA). After the cell protein extracts were obtained, the biotinylated RNA probes to LINC00511 were incubated with cell extracts. Streptavidin beads of 15μL were then added to biotin‐labelled and denatured RNAs and spun at 4°C for 2h. The target sequences were synthesized and biotinylated into biotinylated RNA probes with cell lysis solution. One hour later, the pulled‐down mixture was harvested for analysis. After that, the samples were rotated and incubated overnight at 4°C. Also, centrifugation was carried out after incubation and the RNAs were centrifuged and extracted by TRIzol method and then sent for sequencing. After that, RT‐qPCR was utilized to analyse the mixture of pull‐downs. For DNA pull‐down, DNA pull‐down test kit was employed following the user guide (Gzscbio, Guangzhou, China). Cell proteins were cultured with Biotin‐LINC00511 promoter or No biotin‐LINC00511 promoter as control probe. All pulled‐down mixtures were analysed by qRT‐PCR or Western blot assays.

### 2.19 RNA immunoprecipitation (RIP) assay

Magna RIP™ RNA‐Binding Protein Immunoprecipitation Kit (Millipore) was adopted for the RIP assay. The collected cells were subjected to centrifugation on ice and then they were treated in RIP lysis buffer. Next, cell lysates were incubated with the magnetic beads conjugated with the AGO2 antibody or IgG antibody (negative control). The immunoprecipitated RNA was collected and measured via RT‐qPCR. Bio‐repeats were implemented in triplicate.

### 2.20 Statistical analyses

All assays were conducted in triplicate. Data were exhibited as the mean ± standard deviation (SD). SPSS version 19.0 software (IBM Corp., Armonk, NY) was applied for statistical analysis with *t* test or one‐way ANOVA. The significant levels were specified as *P*‐value below .05.

## 3 RESULTS

### 3.1 LINC00511 was an oncogene and down‐regulated LINC00511 inhibited the progression of GC

**FIGURE 1:**
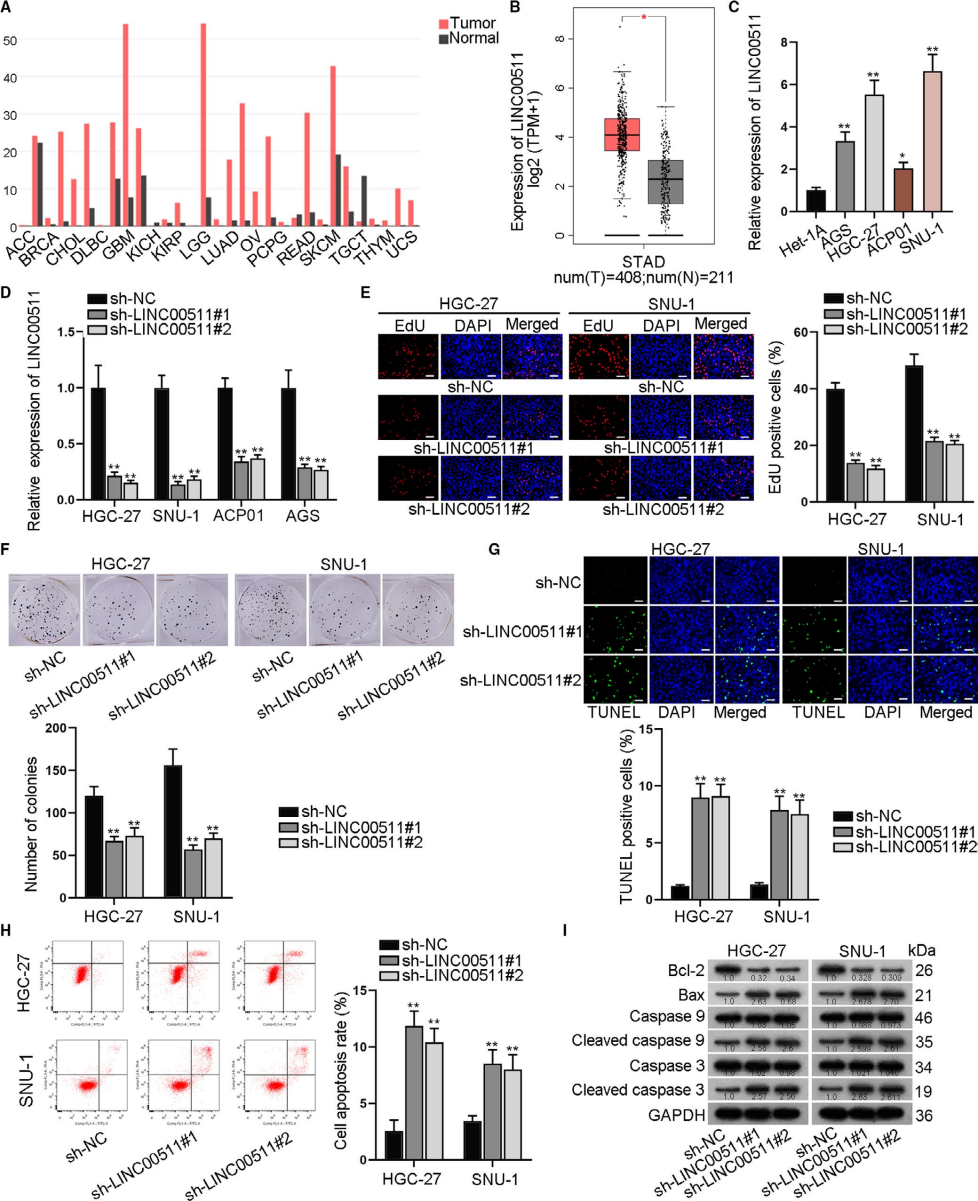
LINC00511 was an oncogene and down‐regulated LINC00511 inhibited the progression of gastric cancer. A‐B, GEPIA database revealed the up‐regulation of LINC00511 in various tissues, including STAD (stomach adenocarcinoma). C, We adopted qRT‐PCR assay to detect LINC00511 expression in GC cells and in normal gastric epithelial cells. D, The interference efficiency of LINC00511 was verified. E‐F, Proliferation ability of GC cells by knockdown of LINC00511 was verified. G‐H, TUNEL and flow cytometry assays were taken to indicate the apoptotic ability of GC cells transfected with sh‐LINC00511#1/2. I, Western blot analysis detected relevant protein levels by LINC00511 knockdown. *P < .05 and **P < .01

As predicted by GEPIA (http://gepia.cancer‐pku.cn/) database, LINC00511 was up‐regulated in multiple cancer tumours (Figure 1A). Based on TCGA data, in stomach adenocarcinoma tissue samples, LINC00511 expression was up‐regulated (Figure 1B). As shown by the result, the expression of LINC00511 in GC cells (AGS, HGC‐27, ACP01, SNU‐1) was higher than that in normal gastric epithelial Het‐1A cell line (Figure 1C). Next, we explored the function of LINC00511 on GC cell proliferation and apoptosis. LINC00511 was firstly knocked down in GC cells and the interference efficiency was detected (Figure 1D). Among the GC cells, HGC‐27 and SNU‐1 cells were kept for following studies due to the better interference efficiency. Then, cell proliferation assays including EdU and colony formation assays disclosed that LINC00511 silencing could lead to the reduction of GC cell proliferative ability (Figure 1E‐F). TUNEL and flow cytometry analysis disclosed that the silencing of LINC00511 could enhance GC cell apoptotic ability (Figure 1G‐H). Furthermore, we conducted Western blot analysis to detect the expression level of apoptosis‐associated proteins and found that expression of Bcl‐2 decreased, while the expression of Bax, cleaved caspase 9 and cleaved caspase 3 increased in LINC00511‐silenced GC cells (Figure 1I). Thus, we concluded that the down‐regulation of LINC00511 suppressed the progression of gastric cancer cells.

### 3.2 LINC00511 promoted GC cell migration, EMT, stemness and GC tumour growth

**FIGURE 2:**
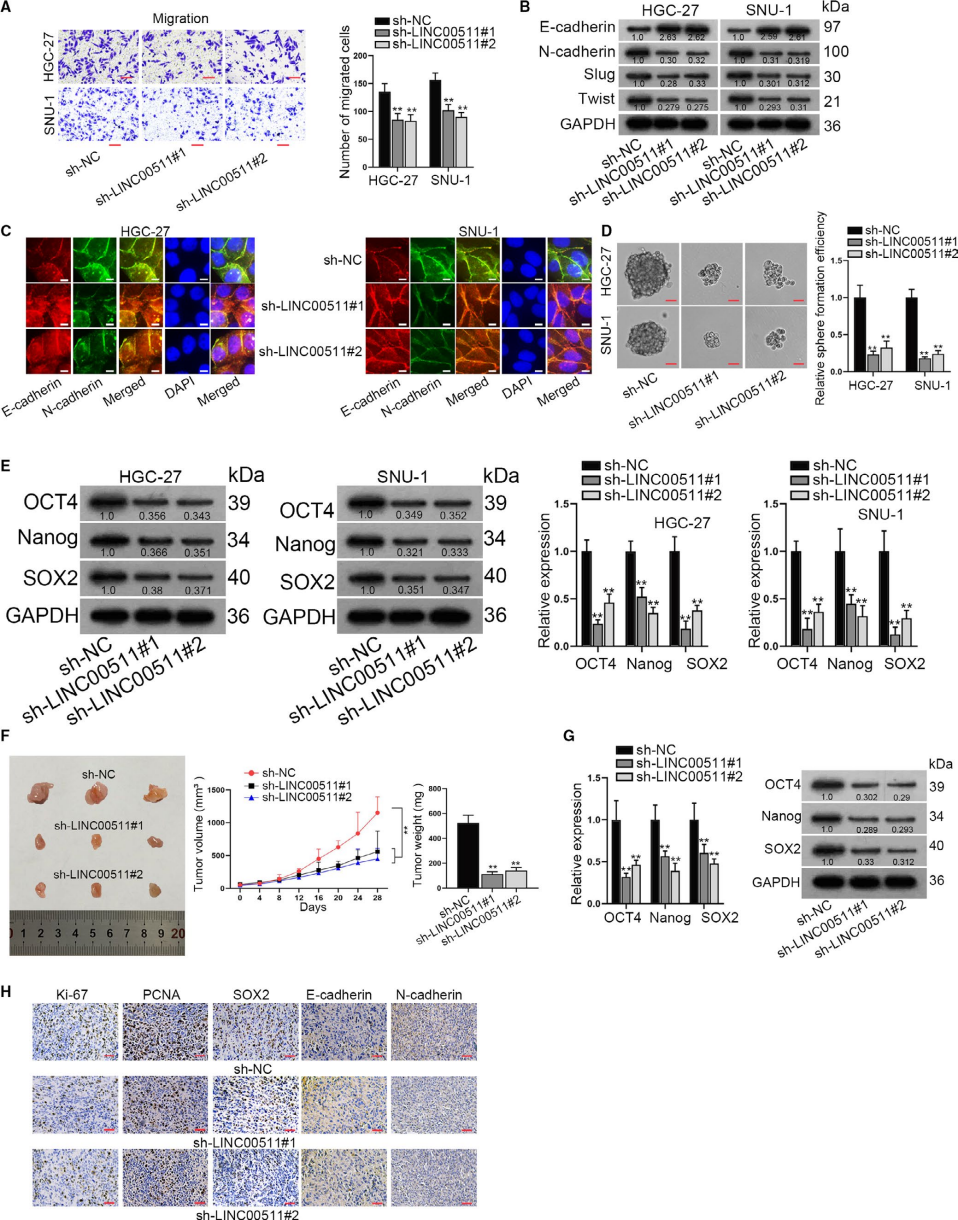
LINC00511 facilitates GC cell migration, EMT, stemness and GC tumour growth. A, Transwell assay detected number of migrated GC cells by knockdown of LINC00511. B, Western blot assay was conducted to detect the expression of relevant proteins in LINC00511‐silenced cells. C, Immunofluorescence staining detected E‐cadherin and N‐cadherin positivity by LINC00511 depletion. D, Sphere formation assay was implemented to detect sphere formation efficiency in GC cells. E, qRT‐PCR and Western blot assays detected the mRNA and protein levels of stemness biomarkers including OCT4, Nanog and SOX2 in GC cells. F, Volume and weight of xenograft tumours by LINC00511 depletion were demonstrated. G, We adopted qRT‐PCR and Western blot assays to detect the protein and mRNA levels of stemness biomarkers in vivo. H, Immunohistochemical staining assay revealed Ki‐67, PCNA, SOX2, E‐cadherin and N‐cadherin positivity by LINC00511 knockdown in vivo. **P < .01

In this section, we conducted a group of functional assays to analyse the mechanism of LINC00511 in GC cell progression. As was shown in Transwell assay, number of migrated cells was decreased after the silencing of LINC00511 in GC cells (Figure 2A). Then, the expression of E‐cadherin, N‐cadherin, Slug and Twist was detected. The results disclosed that the expression level of E‐cadherin was increased, while that of N‐cadherin, Slug and Twist decreased in LINC00511‐down‐regulated GC cells (Figure 2B). IF staining assay revealed that E‐cadherin positivity was enhanced, while N‐cadherin positivity was reduced by LINC00511 depletion in GC cells (Figure 2C). Next, sphere formation assay was implemented and the results revealed that sphere formation efficiency was decreased after the transfection of sh‐LINC00511 in GC cells (Figure 2D). Besides, the expression of stemness biomarkers of OCT4, SOX2 and Nanog was decreased at protein and mRNA level (Figure 2E).

Subsequently, the nude mice xenograft model was established. According to the result of the measurement, both volume and weight of xenograft tumours were decreased after LINC00511 silencing (Figure 2F). Expression of stemness biomarkers in xenograft tumours decreased after the knockdown of LINC00511 at both mRNA and protein levels (Figure 2G). Immunohistochemical staining assay revealed that the positivity of Ki‐67, PCNA, SOX2 and N‐cadherin decreased, while that of E‐cadherin enhanced (Figure 2H). All these data indicated that LINC00511 promoted GC cell migration, EMT and stemness in vitro and facilitated GC tumour growth in vivo.

### 3.3 LINC00511 recruited EZH2 to PTEN promoter and facilitated methylation of PTEN promoter

**FIGURE 3:**
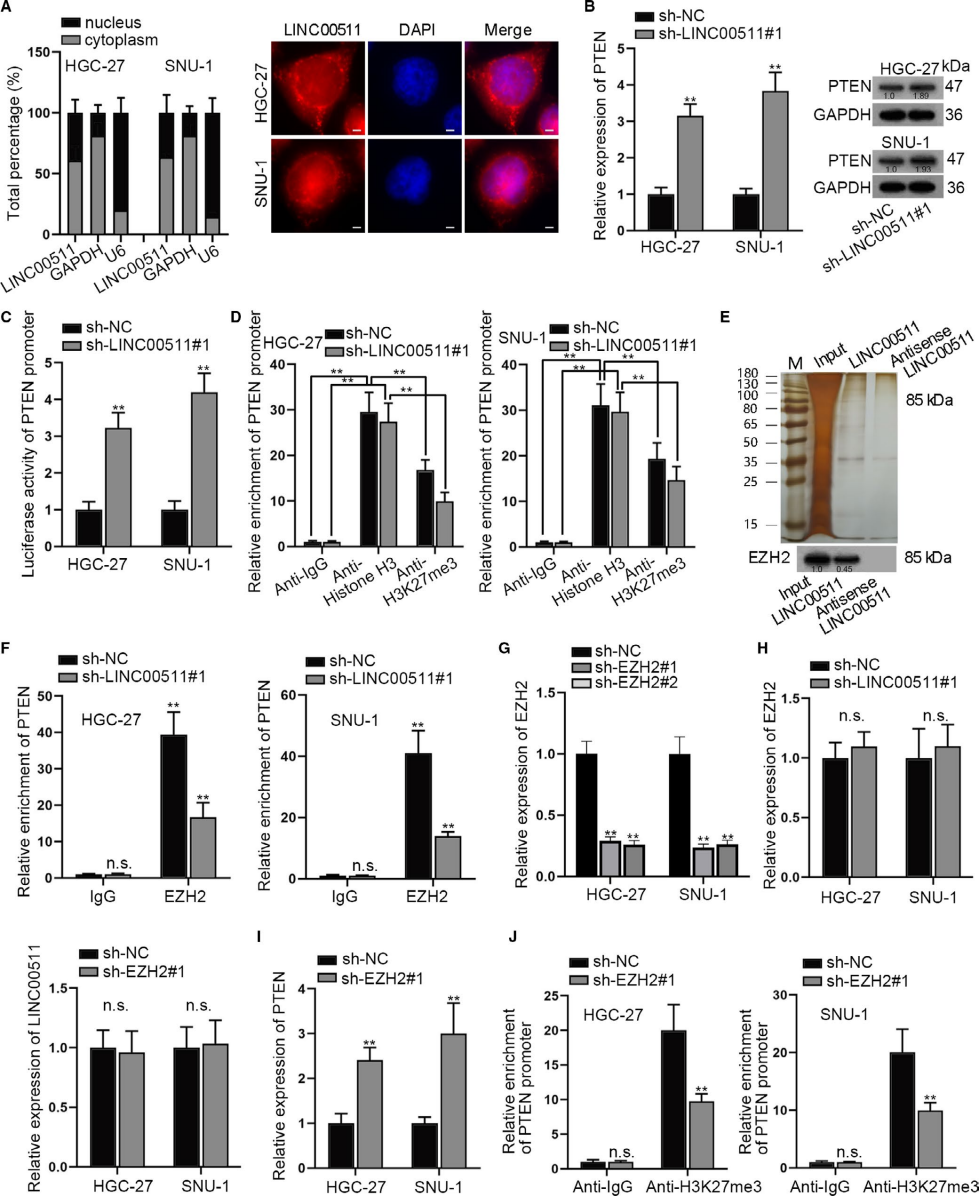
LINC00511 recruited EZH2 to PTEN promoter and facilitated methylation of PTEN promoter. A, Subcellular fraction assay and FISH assay detected subcellular location of LINC00511 in GC cells. B, We adopted qRT‐PCR and Western blot assays to detect mRNA and protein levels of PTEN by LINC00511 knockdown. C, The luciferase activity of PTEN promoter in LINC00511‐silenced cells was detected. D, ChIP assay was conducted to detect the enrichment of PTEN promoter in GC cells after transfection with sh‐NC or sh‐LINC00511#1. E, Silver staining and Western blot disclosed that EZH2 protein was pulled down by LINC00511. F, RIP assay revealed the enrichment of PTEN. G, The depletion efficiency of sh‐EZH2 was detected by RT‐qPCR. H, Influence of LINC00511 depletion on EZH2 and that of EZH2 depletion on LINC00511 was evaluated. I, qRT‐PCR examined the influence of EZH2 depletion on PTEN. J, ChIP assay of PTEN promoter enriched in anti‐IgG and anti‐H3K27me3 in GC cells after transfection with sh‐NC or sh‐EZH2#1. **P < .01. The symbol ‘n.s’ represents no significance

It was widely reported that lncRNAs regulate downstream signalling pathways in cancer development. Therefore, our present study aimed at detecting the influence of LINC0051 on Wnt, PI3K/AKT and Hedgehog pathways. It is revealed in Figure S1A that LINC0051 depletion caused decreased expression of p‐PI3K and p‐AKT in GC cells. Meanwhile, LINC0051 depletion had no effects on Wnt and Hedgehog pathways. Besides, subcellular fraction and FISH assays disclosed that LINC00511 was distributed in both nucleus and cytoplasm in GC cells, indicating that LINC00511 regulated GC cells at both transcriptional and post‐transcriptional levels (Figure 3A). Since PTEN was an up‐stream factor of PI3K/AKT pathway, we next detected the influence of LINC0051 on PTEN expression. It was revealed by qRT‐PCR and Western blot assays that LINC0051 depletion caused increased expression of PTEN (Figure 3B). Besides, it was disclosed that the luciferase activity of PTEN promoter was increased by LINC00511 silencing (Figure 3C). After that, ChIP assay was carried out. Results revealed that when LINC0051 was silenced, binding ability of PTEN promoter and H3K27me3 modification reduced (Figure 3D). Subsequently, we aimed at figuring out which gene could bind to LINC0051. As was shown in RNA pull‐down assay, EZH2 was greatly pulled down by LINC00511, while no obvious genes were pulled down by antisense LINC00511 (Figure 3E). The following RIP assay disclosed that PTEN was greatly enriched in EZH2 group than in IgG group. What's more, when LINC00511 was silenced, PTEN pulled down by EZH2 was remarkably reduced compared with that of sh‐NC group (Figure 3F). Then, we explored the function of EZH2 in GC cells. All the functional assays revealed that EZH2 silencing (Figure S1B) promoted the apoptosis of GC cells (Figure S1D) while inhibiting the cell proliferation (Figure S1C), migration (Figure S1E), EMT (Figure S1F) and stemness (Figure S1G). Then, the interfering efficiency of EZH2 was detected by qPCR in HGC‐27 and SNU‐1 cells (Figure 3G). After that, it was verified that LINC00511 depletion had no obvious effects on EZH2 expression and vice versa (Figure 3H). Next, it was revealed by qRT‐PCR assay that PTEN expression was enhanced by the silencing of EZH2 (Figure 3I). ChIP assay further verified that enrichment of PTEM promoter in anti‐H3K27me3 was reduced when EZH2 was knocked down (Figure 3J). To conclude, LINC00511 recruited EZH2 to PTEN promoter and facilitated the methylation of PTEN promoter.

### 3.4 PTEN partially rescued the effects of LINC00511 on GC cells

**FIGURE 4:**
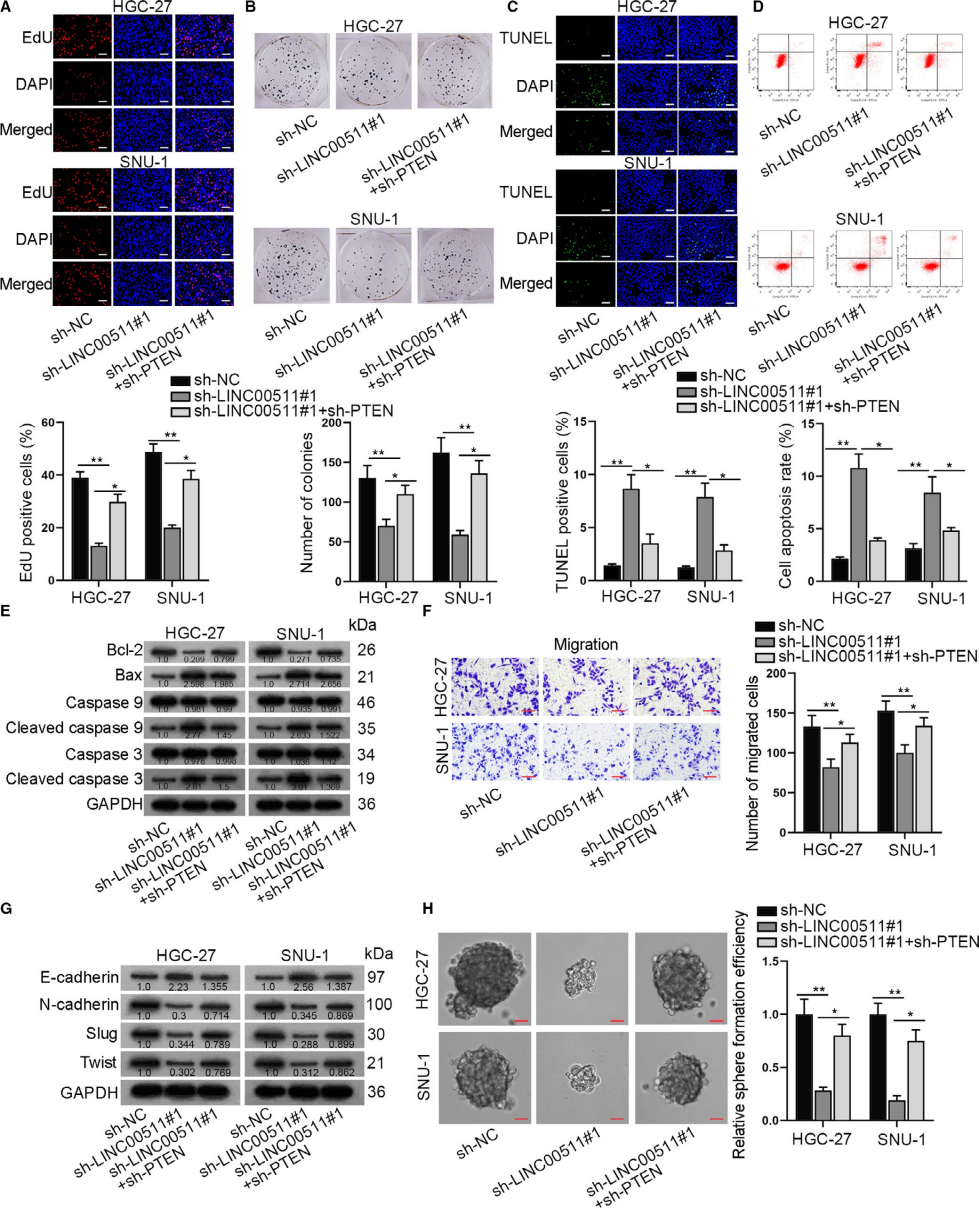
PTEN partially rescued effects of LINC00511 in GC cells. A‐B, EdU and colony formation assay revealed GC cell proliferative ability in sh‐LINC00511 and sh‐LINC00511+sh‐PTEN group. C‐E, TUNEL, flow cytometry assay and Western blot analysis indicated apoptosis ability of GC cells in sh‐LINC00511 and sh‐LINC00511+sh‐PTEN group. F‐H, Transwell, Western blot analysis of EMT‐related proteins and sphere formation assay revealed GC cell migration, EMT and stemness characteristic in sh‐LINC00511 and sh‐LINC00511+sh‐PTEN group. *P < .05 and **P < .01

Since we have verified the regulatory function of LINC00511 on PTEN, we next explored whether PTEN exerted its biological functions on LINC00511‐mediated GC progression. As was demonstrated in EdU and colony formation assays, PTEN depletion partially reversed the suppressive effect of LINC00511 depletion on the proliferative ability of GC cells (Figure 4A‐B). TUNEL assay and flow cytometry analysis were conducted to examine the effect of LINC00511 down‐regulation on GC cell apoptosis under different transfection groups. The apoptosis rate of GC cells was enhanced by silencing LINC00511, which was partially reversed by the cotransfection of sh‐PTEN (Figure 4C‐D). Then, Western blot analysis was conducted to detect apoptosis‐associated proteins under different transfection conditions. We found that compared with NC group, the protein level of Bcl‐2 was declined in the sh‐LINC00511#1 group, while was recovered in the sh‐LINC00511#1+sh‐PTEN group. Meanwhile, the enhanced protein level of Bax, cleaved caspase 9 and cleaved caspase 3 in the sh‐LINC00511#1 group was all reduced in the sh‐LINC00511#1+sh‐PTEN group (Figure 4E). The above data illustrated that the enhanced apoptosis caused by silenced LINC00511 was partially restored by the cotransfection of sh‐PTEN. In addition, as it could be seen from Transwell assay that LINC00511 depletion could suppress the migratory ability of GC cells, while PTEN silencing could reverse this effect (Figure 4F). Western blot analysis of EMT‐related proteins also showed that down‐regulating PTEN could countervail the suppressive effect of LINC00511 silencing on the EMT process of GC cells (Figure 4G). Sphere formation assay was conducted and demonstrated that silenced PTEN could counteract the effect of LINC00511 depletion on the stemness of GC cells in a partial way (Figure 4H). In a word, PTEN depletion partially counteracted LINC00511‐mediated proliferation, apoptosis, migration, EMT and stemness of GC cells.

### 3.5 LINC00511 served as a ceRNA to up‐regulate SOX4 via sponging miR‐195‐5p

**FIGURE 5:**
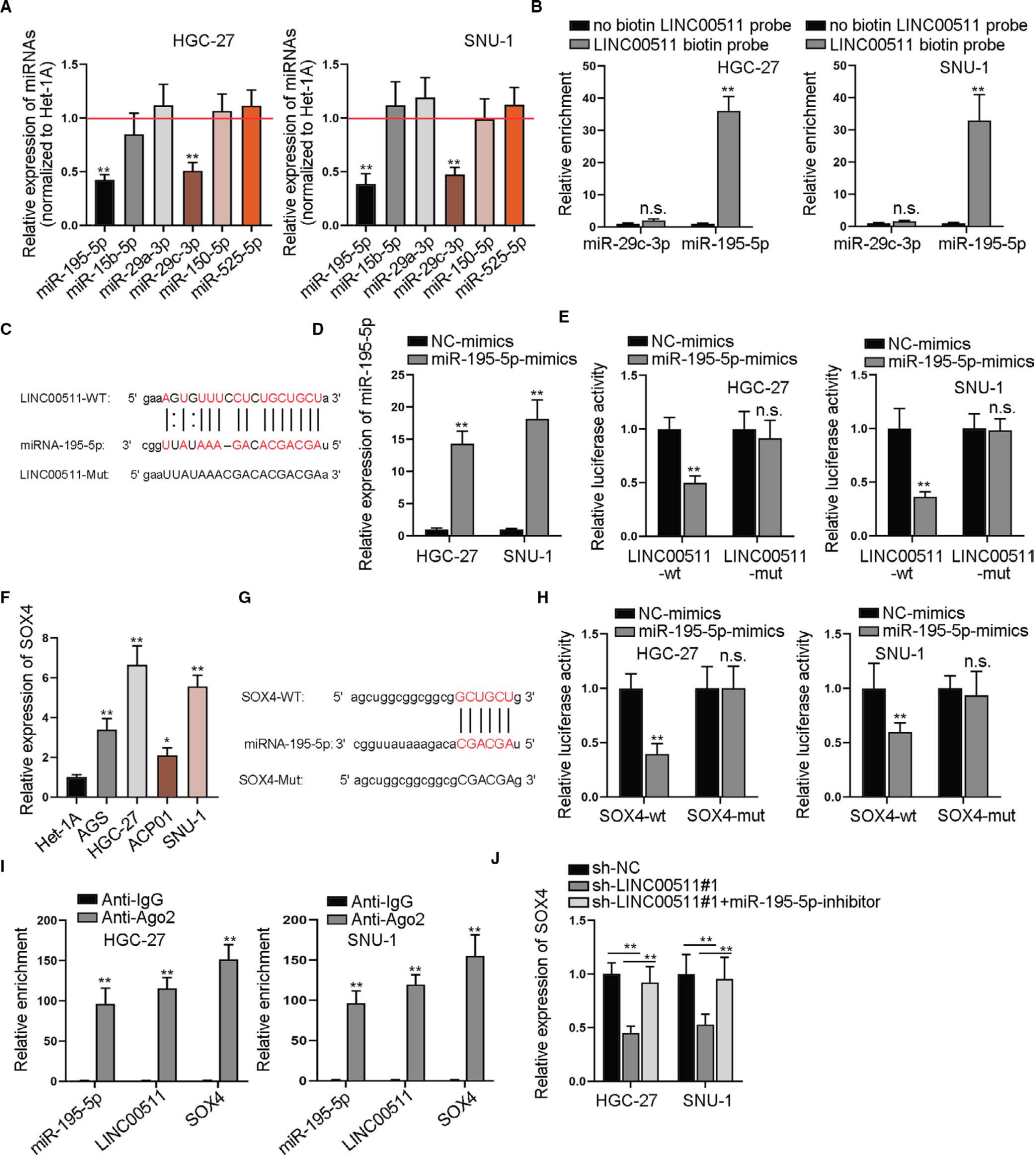
LINC00511 served as a ceRNA to up‐regulate SOX4 via sponging miR‐195‐5p. A, We adopted qRT‐PCR assay to disclose the relative expression of different miRNAs in GC cells compared to normal cells. B, RNA pull‐down assay tested relative enrichment of miR‐29c‐3p and miR‐195‐5p pulled down by biotinylated LINC00511. C, ENCORI database predicted the binding sites between LINC00511 and miR‐195‐5p. D, We detected miR‐195‐5p expression in GC cells transfected with miR‐195‐5p‐mimics through qRT‐PCR assay. E, Luciferase activity of wild‐type and mutant LINC00511 vectors was detected. F, We utilized qRT‐PCR assay to detect SOX4 expression in GC cells and in normal cells. G, Binding sites between SOX4 and miR‐195‐5p were revealed based on ENCORI database. H, The luciferase activity of wild‐type and mutant LINC00511 vectors was detected. I, RIP assay of miR‐195‐5p, LINC00511 and SOX4 in cells treated with antibodies against IgG and Ago2. J, We adopted qRT‐PCR assay to detect the relative expression of SOX4 in GC cells in different groups. *P < .05 and **P < .01. The symbol ‘n.s’ represents no significance

Since the above studies manifested that PTEN partially rescued the effects of LINC00511 on GC cells, and the FISH assay above showed that LINC00511 was also distributed in cytoplasm, we speculated that LINC00511 could exert certain regulatory functions at the post‐transcriptional level. As the ceRNA pattern represents a typical post‐transcriptional mechanism, therefore, we decided to study the potential ceRNA pattern of LINC00511 in GC development. According to ENCORI (http://starbase.sysu.edu.cn/) database, 6 potential miRNAs which might bind to LINC00511 were sifted out (selection condition: 10 cancer types in Pan‐cancer). After the qRT‐PCR assay was conducted, only miR‐195‐5p and miR‐29c‐3p were down‐regulated in GC cells compared with that in the normal Het‐1A cell line (Figure 5A). Then, we constructed biotinylated LINC00511 for RNA pull‐down assay to determine the target miRNA. It was revealed that only miR‐195‐5p was obviously pulled down by biotinylated LINC00511, while no obvious change was seen in miR‐29c‐3p (Figure 5B). Therefore, miR‐195‐5p was kept for further studies. The putative binding sites of wild/mutant LINC00511 and miR‐195‐5p are demonstrated in Figure 5C. After that, we enhanced miR‐195‐5p expression adopted qRT‐PCR assay to detect the overexpression efficiency (Figure 5D). Then, luciferase reporter assay was carried out and it was shown by the result that the luciferase activity in LINC00511‐wt group was decreased upon miR‐195‐5p overexpression, indicating that LINC00511 could bind to miR‐195‐5p at the predicted sites (Figure 5E). Next, we wanted to explore the downstream target of miR‐195‐5p. Based on ENCORI database, we identified that SOX4, a stemness‐associated target, was the potential target for miR‐195‐5p. According to the result shown by qRT‐PCR, SOX4 was verified to be significantly up‐regulated in GC cells (Figure 5F). Since it was verified that LINC00511 deletion could cause the down‐regulation of stemness biomarkers, functional experiments were adopted to examine the effects of SOX4 silencing on the biological behaviours of GC cells. At first, we identified the knockdown of SOX4 by specific shRNAs (Figure S2A). Results of functional assays showed that silencing SOX4 could repress the proliferation (Figure S2B‐C), migration (Figure S2G), EMT (Figure S2H) and stemness of GC cells while enhancing cell apoptosis (Figure S2D, 2E, 2F). The putative binding sites of wild/mutant SOX4 and miR‐195‐5p are demonstrated in Figure 5G. After that, luciferase reporter assay was conducted and it was discovered that the luciferase activity of wild‐type SOX4 was significantly decreased by miR‐195‐5p mimics, while that of mutant‐type SOX4 was not impacted, which demonstrated that SOX4 could bind to miR‐195‐5p (Figure 5H). Subsequently, RIP assay revealed that LINC00511, miR‐195‐5p and SOX4 were significantly enriched in the RNA‐induced silence complex (RISC) in GC cells, demonstrating the co‐existence among LINC00511, miR‐195‐5p and SOX4 (Figure 5I). Besides, qRT‐PCR was conducted to examine the expression of SOX4 in different transfection groups (sh‐NC, sh‐LINC00511#1 and sh‐LINC00511#1+miR‐195‐5p‐inhibitor). According to the result, SOX4 expression was decreased after the transfection of sh‐LINC00511, while it could be reversed by the cotransfection of miR‐195‐5p‐inhibitor (Figure 5J). Therefore, LINC00511 served as a ceRNA to up‐regulate SOX4 via sponging miR‐195‐5p.

### 3.6 SOX4 overexpression restored LINC00511‐medaited effects on GC cells

**FIGURE 6:**
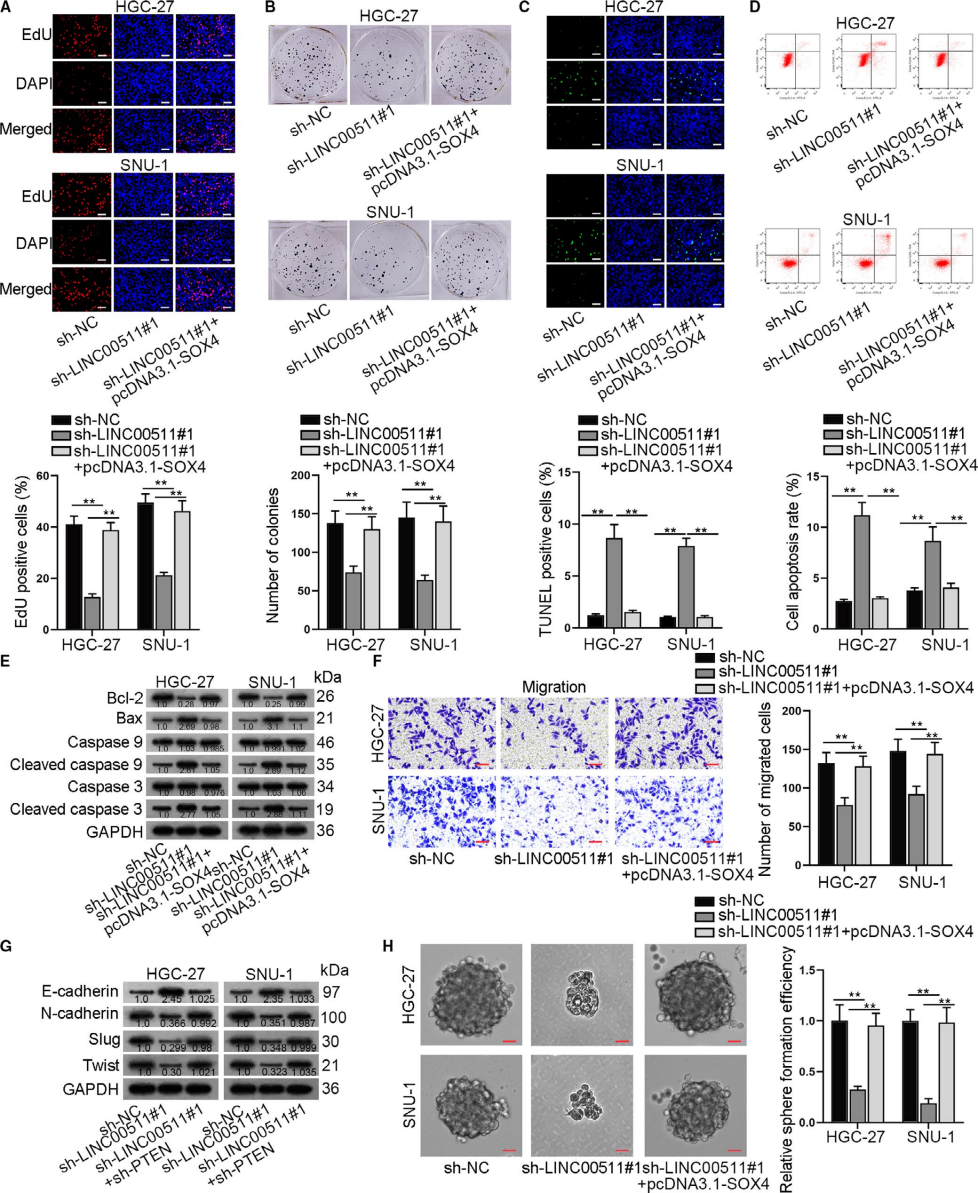
SOX4 overexpression restored LINC00511‐mediated effects on GC cells. A‐B, GC cell migration ability in sh‐LINC00511 and sh‐LINC00511+pcDNA3.1‐SOX4 group was evaluated by EdU and colony formation assays. C‐E, TUNEL, flow cytometry and Western blot analysis of apoptosis‐associated proteins revealed GC cell apoptosis in sh‐LINC00511 and sh‐LINC00511+pcDNA3.1‐SOX4 groups. F‐G, Transwell and Western blot assays were adopted to detect GC cell migration and EMT process in sh‐LINC00511 and sh‐LINC00511+pcDNA3.1‐SOX4 group. H, Sphere formation assay verified sphere formation efficiency in GC cells transfected with sh‐LINC00511 or sh‐LINC00511+pcDNA3.1‐SOX4. **P < .01

After verifying the ceRNA mechanism of LINC00511/miR‐195‐5p/SOX4 and the function of SOX4 in GC cells, rescue assays were utilized to explore the mechanism of miR‐195‐5p/SOX in GC progression. Experimental groups were divided into three groups, namely sh‐NC group, sh‐LINC00511#1 group and sh‐LINC00511#1+pcDNA3.1‐SOX4 group. At first, EdU and colony formation assays were performed to examine the proliferation of GC cells in the above transfection groups, and results showed that GC cell proliferation was reduced by LINC00511 down‐regulation, while was recovered by the cotransfection of pcDNA3.1‐SOX4 (Figure 6A‐B). TUNEL assay, together with flow cytometry analysis, was conducted to examine GC cell apoptosis in different transfection groups, and it was demonstrated that the apoptosis of GC cells enhanced by silencing LINC00511 could be partially reversed by up‐regulating SOX4 (Figure 6C‐D). Western blot assay also suggested that the overexpression of SOX4 could reverse the promoting effect of LINC00511 knockdown on the protein levels of Bax, Cleaved caspase 9 and Cleaved caspase 3 (Figure 6E). As shown by the results, the effects of silenced LINC00511 on GC cell proliferation and apoptosis were rescued by up‐regulation of SOX4. Subsequently, Transwell assay was carried out to examine the migration of GC cells under different transfection conditions, and the result showed that GC cell migration was suppressed by the silencing of LINC00511, while this effect was reversed by the cotransfection of pcDNA3.1‐SOX4 (Figure 6F). The expression level of EMT‐associated proteins also showed that the effect of LINC00511 silencing on the EMT process could be partially reversed by SOX4 overexpression (Figure 6G). Furthermore, the up‐regulation of SOX4 restored the repressive influence caused by LINC00511 depletion on sphere formation efficiency in GC cells (Figure 6H). Thus, we concluded that SOX4 could restore the suppressive effects caused by LINC00511 depletion on the biological behaviours in GC.

### 3.7 SOX4 activated the transcription of LINC00511

**FIGURE 7:**
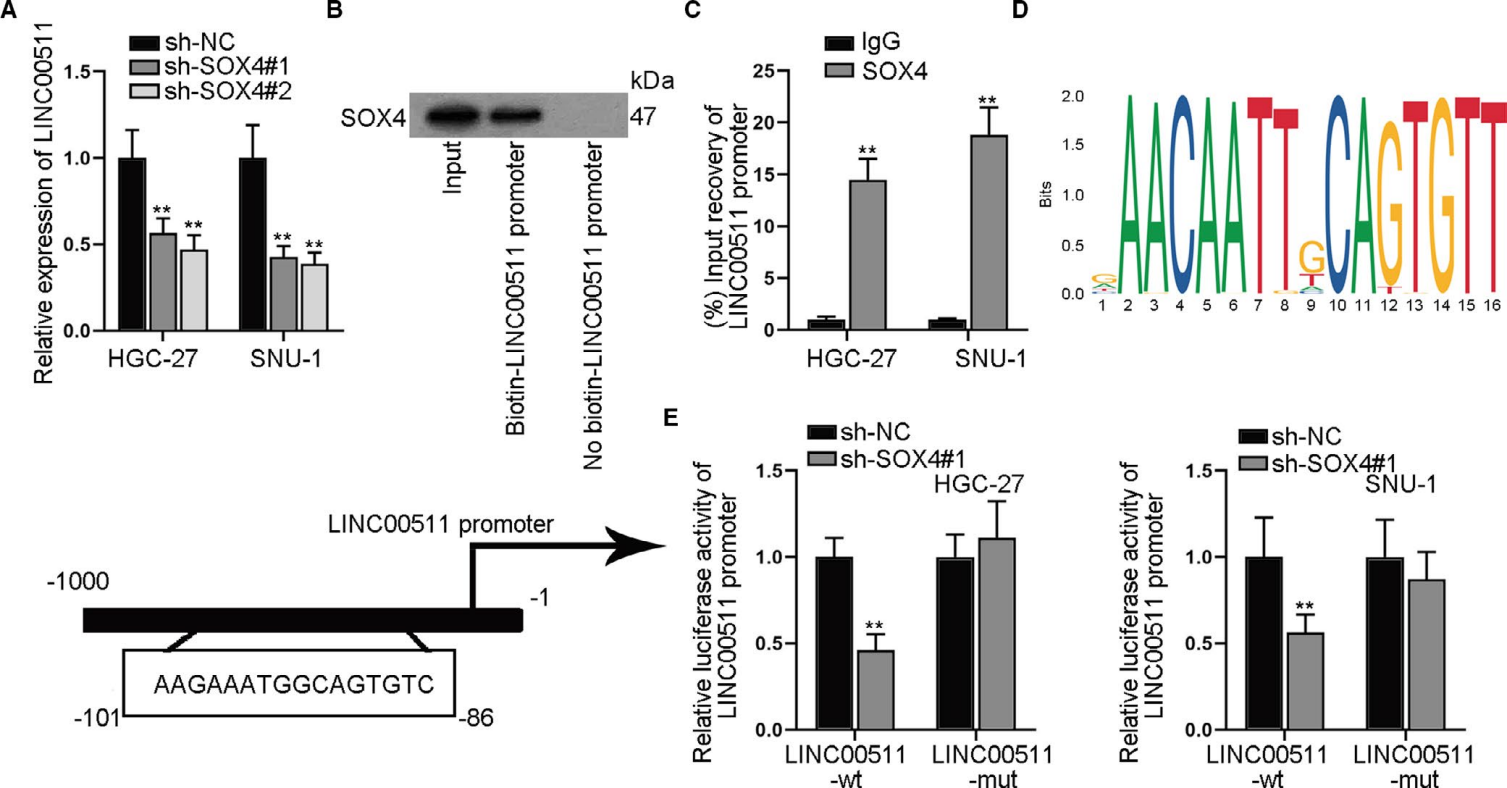
SOX4 activated the transcription of LINC00511. A, We adopted qRT‐PCR assay to detect the relative expression of LINC00511 by SOX4 depletion. B, Western blot assay revealed the binding relationship between SOX4 and LINC00511 promoter. C, ChIP assay verified the affinity between SOX4 and LINC00511 promoter. D, DNA motif and binding sequences of SOX4 on LINC00511 promoter. E, The luciferase activity of wild/mutant LINC00511 promoter by SOX4 depletion was detected. **P < .01

Since SOX4 could restore the LINC00511‐mediated effects on GC cells and SOX4 was reported to serve as a transcription factor, we wondered whether SOX4 activated the transcription of LINC00511. As was shown in qRT‐PCR assay, SOX4 down‐regulation caused reduced expression of LINC00511 (Figure 7A). Also, Western blot and DNA pull‐down assays demonstrated that SOX4 was greatly pulled down by biotin‐labelled LINC00511 promoter (Figure 7B). ChIP assay was conducted, and the affinity between SOX4 and LINC00511 promoter was also verified (Figure 7C). The DNA motif of SOX4 and the predicted binding sites of SOX4 on LINC00511 promoter were demonstrated (Figure 7D). Also, luciferase reporter assay was conducted and it was disclosed that SOX4 could bind to LINC00511 promoter at the predicted sites (Figure 7E). In a word, SOX4 was the transcription factor for LINC00511.

## 4 DISCUSSION

GC represents a common cancer with high morbidity and stands as the second leading cause of cancer‐related mortality.^23^ Therefore, it is of significance to explore more potential biomarkers so as to provide effective therapeutic options for GC treatment. LncRNAs can exert critical functions in the progression of various cancers, and LINC00511 has been proved to be important participant in many cancers, including GC,^14^, ^24^ in which LINC00511 was verified to be an oncogene. Our current study uncovered that LINC00511 was highly expressed in GC cells. By means of loss‐of‐function assays, LINC00511 was verified to promote GC cell proliferation, migration, EMT and stemness while inhibiting cell apoptosis. Also, LINC00511 promoted tumour growth in vivo. Next, we studied the up‐stream mechanism of LINC00511 in GC cells. Associated proteins of PI3K/AKT, hedgehog and Wnt pathway were detected, and the results revealed that LINC00511 could only activate PI3K/AKT pathway which had been widely acknowledged as the oncogenic signalling in GC. According to previous research, UFM1 inhibits the invasion of GC cells by enhancing the ubiquitination level of PDK1 and inactivating PI3K/AKT signalling.^25^ NETO2 increases the phosphorylation of PI3K and AKT to promote the invasion and metastasis of GC.^26^ LncRNA XLOC_006753 accelerates GC cell resistance to 5‐FU and cisplatin via triggering PI3K/AKT/mTOR pathway.^27^

PTEN is the typical up‐stream suppressor of PI3K/AKT pathway. Transgelin 2 directly interacts with PTEN to decrease PTEN expression and promotes paclitaxel resistance as well as the biological behaviours of breast cancer through regulating PI3K/AKT/GSK‐3β pathway.^28^ In addition, PTENP1 hinders breast cancer progression through regulating PTEN/PI3K/AKT pathway.^29^ The down‐regulation of PDZK1 activates PI3K/AKT signalling pathway via phosphorylation of PTEN to facilitate GC cell proliferation.^30^ Here, we unveiled that LINC00511 negatively regulated the expression of PTEN. Moreover, LINC00511 recruited EZH2 protein to the promoter of PTEN and promoted the methylation of PTEN promoter, namely LINC00511 epigenetically repressed PTEN expression. EZH2 was previously revealed as epigenetic H3K27me modifier gene to exert significant role in GC susceptibility.^31^ EZH2 induces EMT and migration and invasion of GC cells by binding to PTEN promoter.^32^ The up‐regulation of lncRNA FOXD2‐AS1 promotes GC tumorigenesis via epigenetically silencing EphB3 through binding to proteins of EZH2 and LSD1.^33^ In the present study, we verified that EZH2 promoted GC cell proliferation and inhibited cell apoptosis. As to reports on methylation of PTEN, EHMT2 contributes to PTEN transcription suppression through epigenetically inhibiting PTEN and facilitates AKT pathway activation to reverse EGFR‐TKI resistance in non‐small‐cell lung cancer.^34^ Besides, DBP, a ubiquitous chemical, was reported to decrease PTEN promoter methylation and increases its transcriptional activity.^35^

Moreover, it was shown by subcellular fraction and FISH assays that LINC00511 existed in both cytoplasm and nucleus of GC cells, which indicated that LINC00511 played a regulatory role in GC cells at the transcriptional and post‐transcriptional levels. Next, we sought to examine how LINC00511 regulated GC cell biological functions at the post‐transcriptional level. According to previous studies, ceRNA mechanism is related to the progression of GC. For example, LINC01133 acts as a ceRNA for miR‐106a‐3p and inhibits GC progression and migration.^36^ MT1JP regulates the progression of GC by playing as a ceRNA to competitively bind to miR‐92a‐3p and regulate FBXW7 expression.^37^ In addition, ILF3‐AS1 functions as a ceRNA to regulate PTBP1 expression by sponging miR‐29a in gastric cancer.^38^ Therefore, mechanism assays were conducted by us to explore the ceRNA mechanism of LINC00511 in GC. LINC00511 was verified to up‐regulate SOX4 by sponging miR‐195‐5p. Also, SOX4 could activate the up‐regulation of LINC00511. MiR‐195‐5p has been reported to inhibit GC cell migration, invasion and GC tumour growth.^39^ Ding L et al have revealed that circ‐DONSON promotes GC progression via recruiting the NURF complex to initiate SOX4 transcription.^40^ SOX4 facilitates TGF‐β‐induced EMT and stem cell characteristics of GC cells.^41^ Besides, SOX4 transcriptionally activates the expression of miR‐17‐92 cluster in prostate cancer progression.^42^ The present study uncovered that LINC00511 promoted GC cell migration, EMT and stemness via the SOX4/miR‐195‐5p axis. The up‐regulation of IGF2‐AS promotes metastasis of gastric adenocarcinoma via serving as an endogenous sponge of miR‐503 to elevate SHOX2 expression.^43^ LINC00346 is transcriptionally activated by KLF5 and MYC and functions as a molecular sponge of miR‐34a‐5p to block its repression on CD44, NOTCH1 and AXL protein translation, thus contributing to GC development.^44^ MT1JP elevates RUNX3 expression through competitively binding miR‐214‐3p to promote tumorigenesis and progression of GC.^45^

To conclude, the present study firstly disclosed that LINC00511 facilitated GC tumour progression at both transcriptional and post‐transcriptional levels. And one graphical abstract has been provided for better understanding. On the one hand, LINC00511 recruited EZH2 to the promoter of PTEN, thus epigenetically repressing PTEN expression and facilitating PI3K pathway activation. On the other hand, LINC00511 was transcriptionally activated by SOX4 and served as a ceRNA of SOX4 by sponging miR‐195‐5p in GC development. We hope that this study can help to provide more valuable therapeutic strategies for the future GC treatment, and more efforts need to be made to further support the clinical outcome of our study.

## CONFLICT OF INTEREST

We declare no conflict of interest.

## AUTHOR CONTRIBUTION

**Qianwei Wang:** Conceptualization (equal); Investigation (equal); Methodology (equal); Project administration (equal); Writing‐original draft (equal); Writing‐review & editing (equal). **Xiang Mao:** Conceptualization (equal); Investigation (equal); Methodology (equal); Project administration (equal); Writing‐original draft (equal); Writing‐review & editing (equal). **Fen Luo:** Formal analysis (equal); Resources (equal); Software (equal); Supervision (equal); Validation (equal). **Jun Wang:** Data curation (equal); Funding acquisition (equal); Visualization (equal).

## Supporting information

Figure S1. The effect of EZH2 knockdown on GC cell growth, migration and stemness. A. Western blot detected associated proteins of Wnt, PI3K/AKT, and Hedgehog pathway in LINC00511‐silenced cells. B. We adopted qRT‐PCR assay to detect the interference efficiency of LINC00511. C‐G. EdU assay (C), TUNEL assay (D), Transwell assay (E), western blot analysis (F) and sphere formation assay (G) revealed effects of EZH2 depletion on GC cell proliferation, apoptosis, migration, EMT and stemness. ^**^ *P* <.01.

Figure S2. The impact of SOX4 silencing on GC cellular processes. A. We adopted qRT‐PCR assay to verify the interference efficiency of SOX4 in GC cells. B‐C. EdU (B), colony formation assays (C) were used to detect the proliferation ability of SOX4‐silenced GC cells. D‐E. TUNEL (C) and flow cytometry analysis were applied to assess the apoptosis rate of GC cells with SOX4 silencing. F. Western blot analysis of apoptosis‐related proteins in SOX4‐down‐regulated GC cells. G. Transwell assays disclosed SOX4 knockdown‐mediated effect on migration. H. Western blot analysis revealed the effect of SOX4 knockdown on EMT markers. I. Sphere formation assays were used to detect the stemness characteristic of SOX4‐down‐regulated GC cells. ^**^ *P* <.01.

## Data Availability

The data that support the findings of this study are available on request from the corresponding author.

## References

[1] Bray F , Ferlay J , Soerjomataram I , Siegel RL , Torre LA , Jemal A . Global cancer statistics 2018: GLOBOCAN estimates of incidence and mortality worldwide for 36 cancers in 185 countries. CA Cancer J Clin. 2018;68:394‐424. 30207593 10.3322/caac.21492

[2] Waddell T , Verheij M , Allum W , Cunningham D , Cervantes A , Arnold D . Gastric cancer: ESMO–ESSO–ESTRO Clinical Practice Guidelines for diagnosis, treatment and follow‐up. Ann Oncol. 2013;6:vi57‐vi63. 10.1093/annonc/mdt344 24078663

[3] Ohtsu A , Ajani JA , Bai YX , et al. Everolimus for previously treated advanced gastric cancer: results of the randomized, double‐blind, phase III GRANITE‐1 study. J Clin Oncol. 2013;31:3935‐3943. 24043745 10.1200/JCO.2012.48.3552 PMC5950503

[4] Fatica A , Bozzoni I . Long non‐coding RNAs: new players in cell differentiation and development. Nat Rev Genet. 2014;15:7‐21. 24296535 10.1038/nrg3606

[5] Wang H , Wu M , Lu Y , et al. LncRNA MIR4435‐2HG targets desmoplakin and promotes growth and metastasis of gastric cancer by activating Wnt/beta‐catenin signaling. Aging. 2019;11:6657‐6673. 31484163 10.18632/aging.102164 PMC6756883

[6] Liang Y , Zhang CD , Zhang C , Dai DQ . DLX6‐AS1/miR‐204‐5p/OCT1 positive feedback loop promotes tumor progression and epithelial–mesenchymal transition in gastric cancer. Gastric Cancer. 2020;23(2):212‐227 31463827 10.1007/s10120-019-01002-1

[7] Xie M , Ma T , Xue J , et al. The long intergenic non‐protein coding RNA 707 promotes proliferation and metastasis of gastric cancer by interacting with mRNA stabilizing protein HuR. Cancer Lett. 2019;443:67‐79. 30502359 10.1016/j.canlet.2018.11.032

[8] Marchese FP , Huarte M . Long non‐coding RNAs and chromatin modifiers: their place in the epigenetic code. Epigenetics. 2014;9:21‐26. 24335342 10.4161/epi.27472 PMC3928181

[9] Liu YW , Xia R , Lu K , et al. LincRNAFEZF1‐AS1 represses p21 expression to promote gastric cancer proliferation through LSD1‐Mediated H3K4me2 demethylation. Mol Cancer. 2017;16:39. 28209170 10.1186/s12943-017-0588-9 PMC5314465

[10] Gu P , Chen X , Xie R , et al. lncRNA HOXD‐AS1 Regulates Proliferation and Chemo‐Resistance of Castration‐Resistant Prostate Cancer via Recruiting WDR5. Mol Ther. 2017;25:1959‐1973. 28487115 10.1016/j.ymthe.2017.04.016 PMC5542640

[11] Chen Z , Wu H , Zhang Z , Li G , Liu B . LINC00511 accelerated the process of gastric cancer by targeting miR‐625‐5p/NFIX axis. Cancer Cell Int. 2019;19:351. 31889903 10.1186/s12935-019-1070-0 PMC6933746

[12] Hu WY , Wei HY , Li KM , Wang RB , Xu XQ , Feng R . LINC00511 as a ceRNA promotes cell malignant behaviors and correlates with prognosis of hepatocellular carcinoma patients by modulating miR‐195/EYA1 axis. Biomed Pharmacother. 2020;121:109642. 31731191 10.1016/j.biopha.2019.109642

[13] Zhu FY , Zhang SR , Wang LH , Wu WD , Zhao H . LINC00511 promotes the progression of non‐small cell lung cancer through downregulating LATS2 and KLF2 by binding to EZH2 and LSD1. Eur Rev Med Pharmacol Sci. 2019;23:8377‐8390. 31646568 10.26355/eurrev_201910_19149

[14] Wang D , Liu K , Chen E . LINC00511 promotes proliferation and invasion by sponging miR‐515‐5p in gastric cancer. Cell Mol Biol Lett. 2020;25:4. 32042282 10.1186/s11658-020-0201-x PMC6998282

[15] Ye Y , Guo J , Xiao P , et al. Macrophages‐induced long noncoding RNA H19 up‐regulation triggers and activates the miR‐193b/MAPK1 axis and promotes cell aggressiveness in hepatocellular carcinoma. Cancer Lett. 2020;469:310‐322. 31705929 10.1016/j.canlet.2019.11.001

[16] Li Z , Liu J , Chen H , et al. Ferritin Light Chain (FTL) competes with long noncoding RNA Linc00467 for miR‐133b binding site to regulate chemoresistance and metastasis of colorectal cancer. Carcinogenesis. 2020;41:467‐477. 31675755 10.1093/carcin/bgz181

[17] Liao K , Lin Y , Gao W , et al. Blocking lncRNA MALAT1/miR‐199a/ZHX1 axis inhibits glioblastoma proliferation and progression. Molecular Therapy Nucleic acids. 2019;18:388‐399. 31648104 10.1016/j.omtn.2019.09.005 PMC6819876

[18] Qiao K , Ning S , Wan L , et al. LINC00673 is activated by YY1 and promotes the proliferation of breast cancer cells via the miR‐515‐5p/MARK4/Hippo signaling pathway. J Exp Clin Cancer Res. 2019;38:418. 31623640 10.1186/s13046-019-1421-7 PMC6796384

[19] Wang LL , Zhang L , Cui XF . Downregulation of long noncoding RNA LINC01419 inhibits cell migration, invasion, and tumor growth and promotes autophagy via inactivation of the PI3K/Akt1/mTOR pathway in gastric cancer. Therap Adv Med Oncol. 2019;11:1758835919874651. 31579114 10.1177/1758835919874651 PMC6759708

[20] Wang B , Zhao CH , Sun G , et al. IL‐17 induces the proliferation and migration of glioma cells through the activation of PI3K/Akt1/NF‐kappaB‐p65. Cancer Lett. 2019;447:93‐104. 30660646 10.1016/j.canlet.2019.01.008

[21] Chen Q , Yang C , Chen L , et al. YY1 targets tubulin polymerisation‐promoting protein to inhibit migration, invasion and angiogenesis in pancreatic cancer via p38/MAPK and PI3K/AKT pathways. Br J Cancer. 2019;121:912‐921. 31631174 10.1038/s41416-019-0604-5 PMC6888832

[22] Wang L , Zhang Z , Yu X , et al. SOX9/miR‐203a axis drives PI3K/AKT signaling to promote esophageal cancer progression. Cancer Lett. 2020;468:14‐26. 31600529 10.1016/j.canlet.2019.10.004

[23] Tan Z . Recent advances in the surgical treatment of advanced gastric cancer: a review. Med Sci Monit. 2019;25:3537‐3541. 31080234 10.12659/MSM.916475 PMC6528544

[24] Huang HG , Tang XL , Huang XS , Zhou L , Hao YG , Zheng YF . Long noncoding RNA LINC00511 promoted cell proliferation and invasion via regulating miR‐124‐3p/EZH2 pathway in gastric cancer. Eur Rev Med Pharmacol Sci. 2020;24:4232‐4245. 32373959 10.26355/eurrev_202004_21003

[25] Lin JX , Xie XS , Weng XF , et al. UFM1 suppresses invasive activities of gastric cancer cells by attenuating the expression of PDK1 through PI3K/AKT signaling. J Exp Clin Cancer Res. 2019;38:410. 31533855 10.1186/s13046-019-1416-4 PMC6751655

[26] Liu JY , Jiang L , He T , et al. NETO2 promotes invasion and metastasis of gastric cancer cells via activation of PI3K/Akt/NF‐kappaB/Snail axis and predicts outcome of the patients. Cell Death Dis. 2019;10:162. 30770791 10.1038/s41419-019-1388-5 PMC6377647

[27] Zeng L , Liao Q , Zou Z , et al. Long Non‐Coding RNA XLOC_006753 promotes the development of multidrug resistance in gastric cancer cells through the PI3K/AKT/mTOR signaling pathway. Cell Physiol Biochem. 2018;51:1221‐1236. 30481766 10.1159/000495499

[28] Liu L , Meng T , Zheng X , et al. Transgelin 2 promotes paclitaxel resistance, migration and invasion of breast cancer by directly interacting with PTEN and activating PI3K/Akt/GSK‐3beta pathway. Mol Cancer Ther. 2019;18(12):2457–2468. 31488699 10.1158/1535-7163.MCT-19-0261

[29] Gao X , Qin T , Mao J , et al. PTENP1/miR‐20a/PTEN axis contributes to breast cancer progression by regulating PTEN via PI3K/AKT pathway. J Exp Clin Cancer Res. 2019;38:256. 31196157 10.1186/s13046-019-1260-6 PMC6567415

[30] Zhao C , Tao T , Yang L , et al. Loss of PDZK1 expression activates PI3K/AKT signaling via PTEN phosphorylation in gastric cancer. Cancer Lett. 2019;453:107‐121. 30930234 10.1016/j.canlet.2019.03.043

[31] Lee SW , Park DY , Kim MY , Kang C . Synergistic triad epistasis of epigenetic H3K27me modifier genes, EZH2, KDM6A, and KDM6B, in gastric cancer susceptibility. Gastric Cancer. 2019;22:640‐644. 30374835 10.1007/s10120-018-0888-9

[32] Gan L , Xu M , Hua R , et al. The polycomb group protein EZH2 induces epithelial‐mesenchymal transition and pluripotent phenotype of gastric cancer cells by binding to PTEN promoter. J Hematol Oncol. 2018;11:9. 29335012 10.1186/s13045-017-0547-3 PMC5769437

[33] Xu TP , Wang WY , Ma P , et al. Upregulation of the long noncoding RNA FOXD2‐AS1 promotes carcinogenesis by epigenetically silencing EphB3 through EZH2 and LSD1, and predicts poor prognosis in gastric cancer. Oncogene. 2018;37:5020‐5036. 29789713 10.1038/s41388-018-0308-y

[34] Wang L , Dong X , Ren Y , et al. Targeting EHMT2 reverses EGFR‐TKI resistance in NSCLC by epigenetically regulating the PTEN/AKT signaling pathway. Cell Death Dis. 2018;9:129. 29374157 10.1038/s41419-017-0120-6 PMC5833639

[35] Li R , Xing QW , Wu XL , et al. Di‐n‐butyl phthalate epigenetically induces reproductive toxicity via the PTEN/AKT pathway. Cell Death Dis. 2019;10:307. 30952838 10.1038/s41419-019-1547-8 PMC6450951

[36] Yang XZ , Cheng TT , He QJ , et al. LINC01133 as ceRNA inhibits gastric cancer progression by sponging miR‐106a‐3p to regulate APC expression and the Wnt/β‐catenin pathway. Mol Cancer. 2018;17:126. 30134915 10.1186/s12943-018-0874-1 PMC6106894

[37] Zhang G , Li S , Lu J , et al. LncRNA MT1JP functions as a ceRNA in regulating FBXW7 through competitively binding to miR‐92a‐3p in gastric cancer. Mol Cancer. 2018;17:87. 29720189 10.1186/s12943-018-0829-6 PMC5930724

[38] Ren ZH , Shang GP , Wu K , Hu CY , Ji T . WGCNA Co‐expression network analysis reveals ILF3‐AS1 functions as a CeRNA to regulate PTBP1 expression by sponging miR‐29a in gastric cancer. Frontiers in Genetics. 2020;11:39. 32117452 10.3389/fgene.2020.00039 PMC7033569

[39] Wang J , Li L , Jiang M , Li Y . MicroRNA‐195 inhibits human gastric cancer by directly targeting basic fibroblast growth factor. Clin Transl Oncol. 2017;19:1320‐1328. 28500362 10.1007/s12094-017-1668-4

[40] Ding L , Zhao Y , Dang S , et al. Circular RNA circ‐DONSON facilitates gastric cancer growth and invasion via NURF complex dependent activation of transcription factor SOX4. Mol Cancer. 2019;18:45. 30922402 10.1186/s12943-019-1006-2 PMC6437893

[41] Peng X , Liu G , Peng H , Chen A , Zha L , Wang Z . SOX4 contributes to TGF‐beta‐induced epithelial‐mesenchymal transition and stem cell characteristics of gastric cancer cells. Genes Diseases. 2018;5:49‐61. 30258935 10.1016/j.gendis.2017.12.005 PMC6147107

[42] Liu H , Wu Z , Zhou H , et al. The SOX4/miR‐17‐92/RB1 axis promotes prostate cancer progression. Neoplasia (New York, NY). 2019;21:765‐776. 10.1016/j.neo.2019.05.007 PMC6593351 31238254

[43] Huang J , Chen YX , Zhang B . IGF2‐AS affects the prognosis and metastasis of gastric adenocarcinoma via acting as a ceRNA of miR‐503 to regulate SHOX2. Gastric Cancer. 2020;23(1):23‐38 31183590 10.1007/s10120-019-00976-2

[44] Xu TP , Ma P , Wang WY , et al. KLF5 and MYC modulated LINC00346 contributes to gastric cancer progression through acting as a competing endogeous RNA and indicates poor outcome. Cell Death Differ. 2019;26:2179‐2193. 30770877 10.1038/s41418-018-0236-y PMC6888833

[45] Xu Y , Zhang G , Zou C , et al. LncRNA MT1JP Suppresses Gastric Cancer Cell Proliferation and Migration Through MT1JP/MiR‐214‐3p/RUNX3 Axis. Cell Physiol Biochem. 2018;46:2445‐2459. 29742512 10.1159/000489651

