# Supplementary figures and images for: LINC00511 promotes gastric cancer progression by regulating SOX4 and epigenetically repressing PTEN to activate PI3K/AKT pathway
Source: J Cell Mol Med. 2021 Aug 24;25(19):9112–27. doi: 10.1111/jcmm.16656 (PMC8500959; doi:10.1111/jcmm.16656)

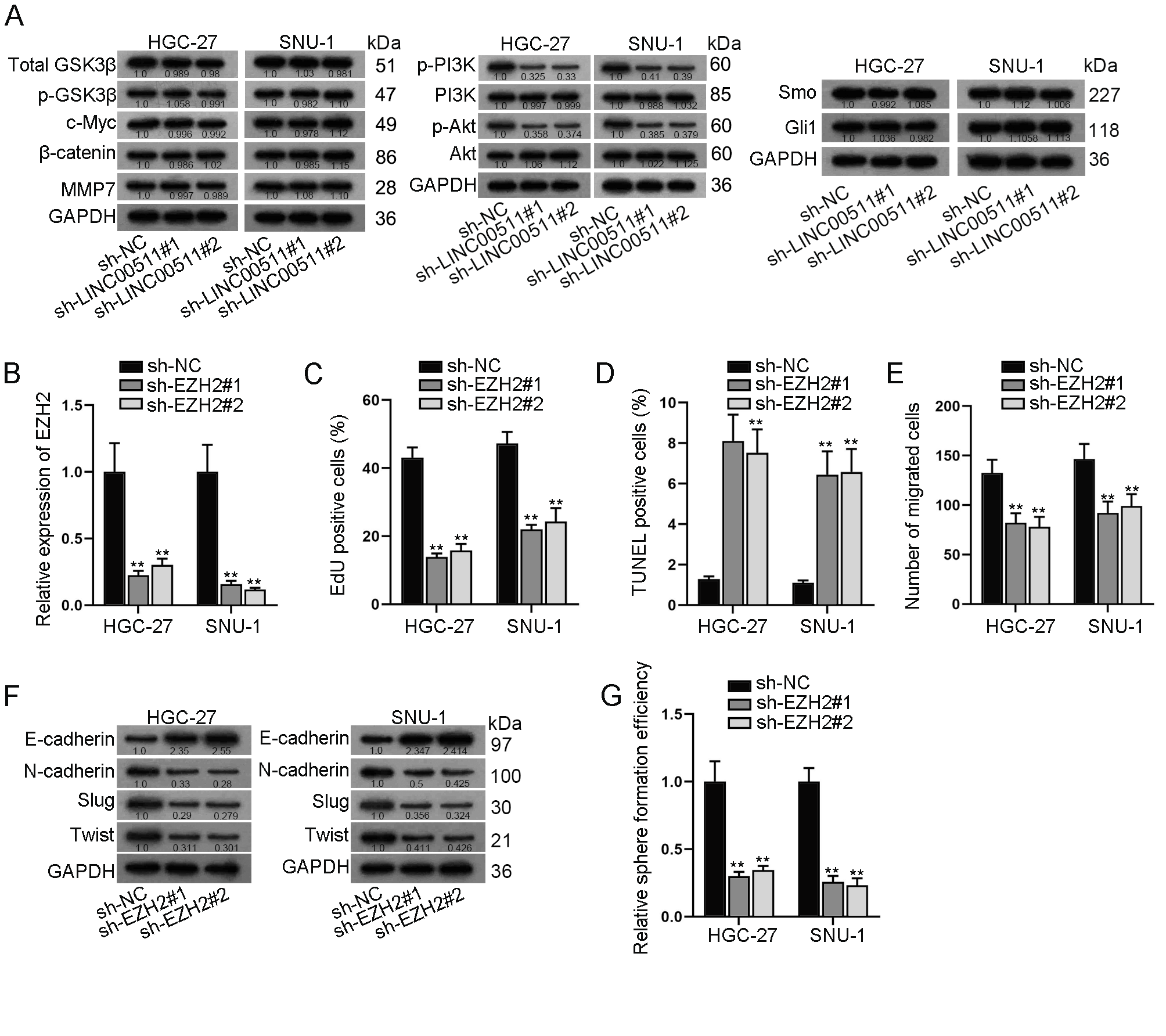

Supplement: Supplementary file 1 — Figure S1. The effect of EZH2 knockdown on GC cell growth, migration and stemness. A. Western blot detected associated proteins of Wnt, PI3K/AKT, and Hedgehog pathway in LINC00511‐silenced cells. B. We adopted qRT‐PCR assay to detect the interference efficiency of LINC00511. C‐G. EdU assay (C), TUNEL assay (D), Transwell assay (E), western blot analysis (F) and sphere formation assay (G) revealed effects of EZH2 depletion on GC cell proliferation, apoptosis, migration, EMT and stemness. ** P <.01. [file JCMM-25-9112-s001.tif]

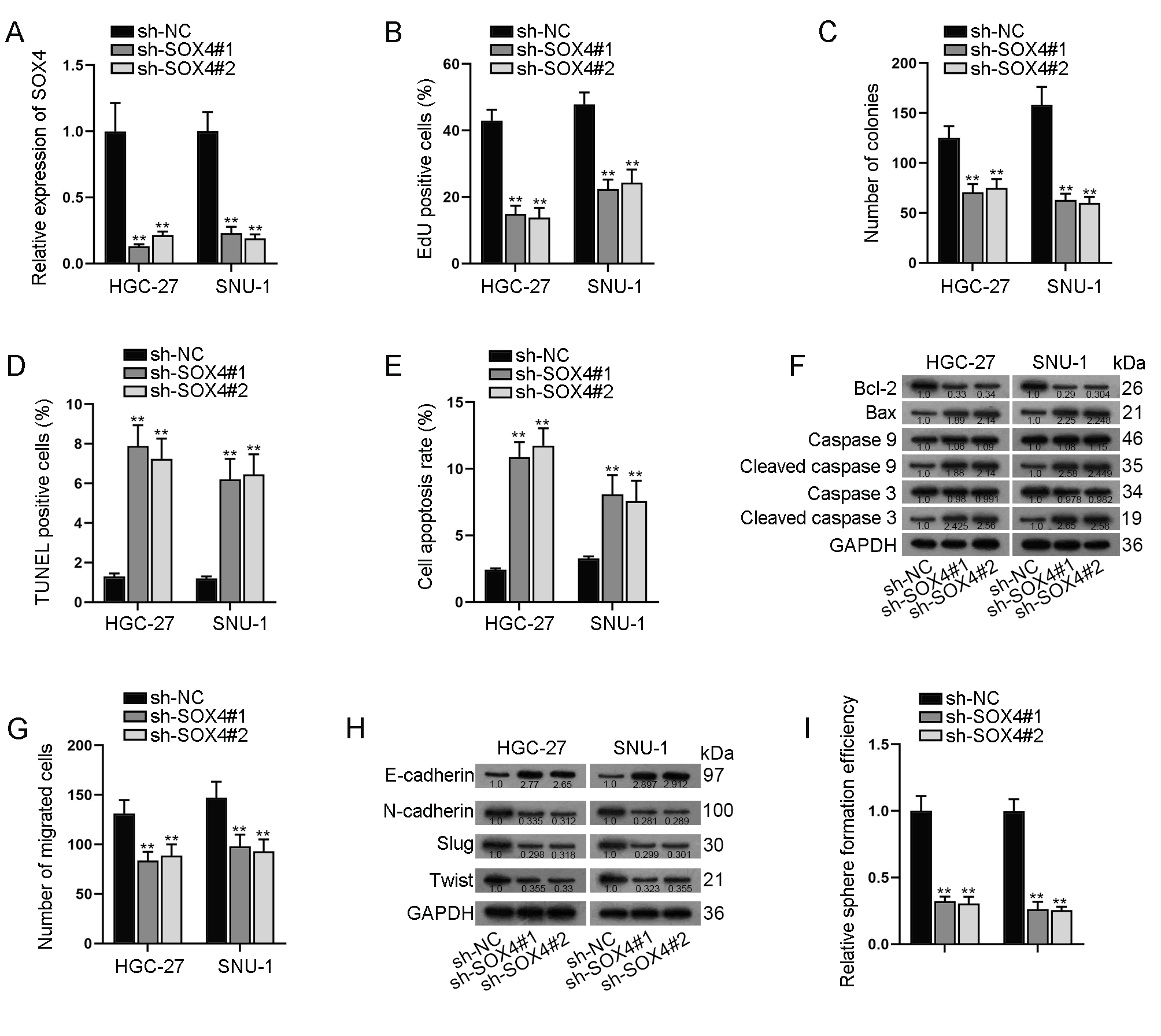

Supplement: Supplementary file 2 — Figure S2. The impact of SOX4 silencing on GC cellular processes. A. We adopted qRT‐PCR assay to verify the interference efficiency of SOX4 in GC cells. B‐C. EdU (B), colony formation assays (C) were used to detect the proliferation ability of SOX4‐silenced GC cells. D‐E. TUNEL (C) and flow cytometry analysis were applied to assess the apoptosis rate of GC cells with SOX4 silencing. F. Western blot analysis of apoptosis‐related proteins in SOX4‐down‐regulated GC cells. G. Transwell assays disclosed SOX4 knockdown‐mediated effect on migration. H. Western blot analysis revealed the effect of SOX4 knockdown on EMT markers. I. Sphere formation assays were used to detect the stemness characteristic of SOX4‐down‐regulated GC cells. ** P <.01. [file JCMM-25-9112-s002.tif]
